# The Efficacy of Roux-en-Y Gastric Bypass in Young-Onset Diabetes Mellitus: A Narrative Review

**DOI:** 10.7759/cureus.67562

**Published:** 2024-08-23

**Authors:** Rishika Bhatnagar, Snehlata Hingway, Dushyant Bawiskar

**Affiliations:** 1 Pathology, Jawaharlal Nehru Medical College, Datta Meghe Institute of Higher Education and Research, Wardha, IND; 2 Sports Medicine, Abhinav Bindra Targeting Performance, ‎Bengaluru, IND

**Keywords:** bariatric surgery, glycemic control, weight loss, roux-en-y gastric bypass, type 2 diabetes mellitus

## Abstract

Type 2 diabetes mellitus (T2DM) in youth is invading the communities because, if not controlled on time, the long-term complications include cardiovascular diseases, nephropathy, neuropathy, and retinopathy that cause immense mortality and morbidity. Lifestyle changes and antidiabetic drugs are considered the foundation of T2DM therapy. However, these adjustments usually do not effectively produce long-term glycemic regulation, especially in patients with obesity of the third and fourth degrees. Bariatric surgery has also been identified as an efficacious intervention for obesity and obesity-related complications such as T2DM. Roux-en-Y gastric bypass (RYGB) has proven to be one of the most effective procedures in causing considerable weight loss and enhancing glycemic changes. This review provides a comprehensive analysis of RYGB in patients with young-onset T2DM regarding the improvement of glycemic control, weight loss, and diabetes comorbidities. RYGB has been established as a practice in the treatment of T2DM and severe obesity. This narrative review underscores the various effects of RYGB, such as enhanced glycemic control, considerable and long-term weight loss, and reduced cardiovascular disease risks. However, the review also points toward the directions and the adverse effects of RYGB regarding metabolic and skeletal health. There are risks of nutritional deficiencies, increased fracture rates, and even relapse to diabetes, which make patient selection, proper pre and postoperative investigation, and critical monitoring.

## Introduction and background

Type 2 diabetes mellitus (T2DM) is one of the most common chronic diseases, characterized by insulin resistance and insufficient insulin production by the beta-cells of the pancreas that result in elevated blood glucose levels. It is widely considered a significant health problem, which is prevalent and on the rise, especially among the youth. T2DM in youth is invading the communities because, if not controlled on time, the long-term complications include cardiovascular diseases, nephropathy, neuropathy, and retinopathy that cause immense mortality and morbidity [1-4]. Lifestyle changes and antidiabetic drugs are considered the foundation of T2DM therapy. However, these adjustments usually do not effectively produce long-term glycemic regulation, especially in patients with third and fourth-degree obesity. Bariatric surgery has been identified as an efficacious intervention for obesity and obesity-related complications such as T2DM. Of the different bariatric operations, Roux-en-Y gastric bypass (RYGB) has proven to be one of the most effective procedures in causing considerable weight loss and enhancing glycemic changes [5,6].

RYGB is a surgical procedure wherein the surgeon develops a small pouch in the stomach, and a section of the small intestine is bypassed. This change of behavior not only limits the consumption of food but directs hormonal changes that promote the effectiveness and volume of insulin and secretion, yielding almost immediate and effective management of glycemia. The effectiveness of RYGB surgery in the treatment of or contribution to the remission or significant amelioration of overweight/obesity-associated T2DM in the adult population is abundantly clear in the literature; however, its implication for young-onset T2DM remains relatively unknown [7,8]. Numerous global medical and surgical organizations have agreed that bariatric surgery should be included in the treatment options for type 2 diabetes in adults, as it has been used for a long time as a therapy for severe obesity. Vertical sleeve gastrectomy (VSG), adjustable gastric banding (AGB), and RYGB are the most often used bariatric procedures in both adults and adolescents. RYGB and VSG are the most successful among them in terms of improving metabolic health and lowering body weight [9].

This review provides a comprehensive analysis of RYGB in patients with young-onset T2DM concerning the improvement of glycemic control, weight loss, and diabetes comorbidities. This review aims to reveal the advantages and possible drawbacks of RYGB as a treatment for youths with T2DM based on the accumulated research findings.

## Review

The efficacy of RYGB for managing T2DM has been extensively studied, with numerous studies demonstrating its benefits. Giudicelli et al. found that RYGB enhances metabolic health and diabetes outcomes in both young and older non-insulin-dependent diabetic adults, emphasizing the importance of timely surgery for better glycemic control and reduced cardiovascular risk [10]. Hage et al. reported that RYGB is more effective than sleeve gastrectomy (SG) for T2DM remission, regardless of baseline characteristics and severity [11].

Girundi et al. concluded that RYGB is highly effective for long-term glycemic control, with 87.6% of patients experiencing T2DM remission 18 months post-surgery [12]. Cheng et al. demonstrated that RYGB is more effective than medical treatment for achieving glycemic control over five years, with a higher probability of maintaining HbA1c levels below 6.5% without medication [13]. Ji et al. showed that RYGB benefits T2DM patients with a body mass index (BMI) under 32.5 kg/m², improving weight loss, glycemic control, and lipid profiles over a six-year follow-up [14].

Nudotor et al. found that RYGB is significantly more effective than VSG for sustained T2DM remission over five years [15]. van Rijswijk et al. reported that both RYGB and one-anastomosis gastric bypass are effective for rapid T2DM remission in severely obese patients [16]. Du et al. confirmed the safety, efficacy, and practicality of laparoscopic RYGB for treating obese Chinese T2DM patients over a three-year follow-up [17]. Zhang et al. found that RYGB is superior to SG for medication decrease and dyslipidemia remission [18].

Sahin et al. highlighted the need to prioritize patient outcomes over economic considerations in non-obese overweight groups undergoing bariatric surgery. Collectively, these studies indicate that RYGB is highly effective for inducing T2DM remission, improving glycemic control, and achieving significant weight loss, outperforming other bariatric procedures such as SG and VSG in most metrics, making it a valuable option for managing T2DM in various patient populations [19]. van de Pas et al. conducted a nationwide cohort study on 2,822 young adults and 24,497 adults, revealing that bariatric surgery, specifically RYGB or SG, is equally safe and effective across age groups, with young adults achieving superior weight loss. Guimarães et al. found that RYGB effectively promotes long-term weight loss and improves obesity-related comorbidities with minimal serious complications in a study of 601 patients. Sharples et al. demonstrated that RYGB significantly improved diabetic control, reduced medication use, and achieved substantial weight loss and cost savings in 52 patients with T2DM [20-22].

Advantages of Roux-en-Y gastric bypass

RYGB offers significant advantages in T2DM by inducing improvements in glucose metabolism and insulin sensitivity, as well as enhancing beta-cell function. Research indicates that RYGB leads to early increases in insulin secretion and sustained improvements in glucose tolerance and insulin sensitivity, which are maintained even seven years post-surgery [23-25]. Furthermore, RYGB triggers changes in beta-cell mass, promoting trans-differentiation into alpha-cell mass due to long-term adaptation of the digestive tract post-surgery, contributing to the resolution of T2DM. Additionally, the effects of the procedure on s-cell function are crucial for the long-term management of T2DM, highlighting the lasting benefits of RYGB in treating this metabolic disorder [26]. RYGB specifically improves insulin sensitivity in the liver, muscle, and fat, as evidenced by increased glucose disposal and decreased endogenous glucose production [27]. Furthermore, RYGB induces marked improvements in glucose tolerance, insulin sensitivity in muscle and liver, and beta-cell function, which are maintained even up to seven years post-surgery [28].

RYGB significantly impacts ghrelin levels, a hormone associated with hunger regulation. Research indicates that both RYGB and laparoscopic sleeve gastrectomy (LSG) lead to notable reductions in serum ghrelin concentrations one-year post-surgery, with decreases of approximately 0.068 pg/mL for RYGB and 0.083 pg/mL for LSG. However, these changes were not statistically different between the two procedures. Additionally, a study on mice demonstrated that while ghrelin levels increased post-surgery, lower ghrelin levels did not affect the metabolic benefits of RYGB, suggesting that other mechanisms may also contribute to weight loss and metabolic improvements. Furthermore, ghrelin modulation has been explored as a potential therapeutic strategy to enhance weight loss outcomes in patients with suboptimal responses to bariatric surgery. Overall, RYGB effectively reduces ghrelin levels, contributing to its role in weight management and metabolic health [29,30].

Disadvantages of Roux-en-Y gastric bypass

Despite initial improvements, many patients relapse within five years due to changes in the beta-cell population and absorptive intestinal effects. This leads to beta-cell exhaustion and a decrease in beta-cell mass. Risks associated with RYGB include higher rates of mortality and morbidity compared to other bariatric procedures, long-term nutritional deficiencies (such as bone demineralization), and potential complications such as ulcers, bowel obstructions, and hypoglycemia syndromes [31-33]. Dumping syndrome is a common issue post-RYGB, characterized by symptoms such as borborygmus, diarrhea, abdominal distention, pain, palpitations, tachycardia, hypotension, sweating, flushing, and fatigue, typically occurring within 30 minutes of eating. A Danish questionnaire-based study by Grabsholt et al. found that 52.4% of 1,429 participants who had RYGB 4.7 years prior reported experiencing one or more dumping syndrome-related symptoms [33,34].

Nutritional deficiencies and altered nutrient absorption are significant concerns after RYGB, necessitating lifelong supplementation of vitamin B12, vitamin D, calcium, iron, and folic acid. Anemia, affecting about 12% of patients at baseline, is the most prevalent clinical symptom of these deficiencies. Additionally, there is concern that RYGB impairs bone metabolism, increasing the risk of fractures, osteoporosis, and osteopenia [35,36]. Table 1 depicts a description of the studies included in the review.

**Table 1:** Description of the studies included in the review. RYGB: Roux-en-Y gastric bypass, T2DM: Type 2 Diabetes Mellitus, HbA1c: Glycated haemoglobin, BMI: Body Mass Index, LDL: Low-density lipoprotein, HDL: High-Density Lipoprotein, OGTT: Oral glucose tolerance test, VSG: vertical sleeve gastrectomy, OAGB: one-anastomosis gastric bypass, SG: Sleeve gastrectomy

Author and year	Participants	Intervention	Outcome measures	Conclusion
Giudicelli et al. (2023) [10]	169 non-insulin-dependent diabetic adults who underwent RYGB. 55 young adults and 114 older adults	To ensure group comparability, young adults were matched one to two with older adults based on sex, BMI, duration of preoperative diabetes, and the American Society of Anesthesiologists score	Primary: Diabetes remission, focusing on rates and timing of remission post-surgery. Secondary: Percent weight change, loss to follow-up, and all-cause adverse events	The results support the efficacy of RYGB in enhancing metabolic health and diabetes outcomes in both young and older non-insulin-dependent diabetic adults, emphasizing the importance of timely metabolic surgery for better glycemic control and reduced cardiovascular risk
Hage et al. (2024) [11]	1,149 patients with T2DM who underwent either RYGB or SG	The study evaluated the rates of T2DM remission, insulin use, changes in HbA1c levels, and diabetes medication usage for both RYGB and SG patients to determine the effectiveness of each procedure in managing T2DM	Diabetes remission comparison, weight change, and adverse events and long-term metabolic outcomes	RYGB has higher efficacy for T2DM remission than SG, regardless of baseline characteristics, T2DM severity, weight loss, or follow-up duration
Girundi (2016) [12]	468 patients with T2DM who underwent RYGB	Patients’ glycemic profiles were assessed at specific intervals after surgery, including the 3rd, 6th, 9th, 12th, and 18th months	Remission of T2DM	The study concluded that RYGB was highly effective in achieving long-term glycemic control, with 87.6% of patients experiencing remission of type 2 diabetes 18 months after the surgery
Cheng et al. (2022) [13]	100 adults with T2DM	Throughout the five-year study, participants were periodically examined to evaluate the results and monitor for any side effects	HbA1c levels of less than 6.5%, weight loss, changes in cardiovascular risk factors, quality of life, and adverse events	Over 5 years, in adults with type 2 diabetes mellitus and a BMI between 27 and 32 kg/m², RYGB was more effective than the medical treatment for achieving glycemic control. Individuals undergoing RYGB surgery had a higher probability of attaining HbA1c levels below 6.5% without requiring diabetes medication in comparison to those undergoing medical treatment
Ji et al. (2020) [14]	52 patients with T2DM	Data were collected at baseline, at 3, 6, and 1, 2, 3, 4, 5, and 6 years after surgery to monitor changes in the measured parameters over time	Throughout the 6-year study period, alterations in lipid profiles (triglycerides, total cholesterol, LDL, HDL, BMI, HbA1c levels, OGTT results, and the remission or improvement of T2DM) were noted	Results indicate that T2DM patients with a BMI < 32.5 kg/m^2^ might benefit from RYGB, which can result in notable weight loss, better glycemic control, and positive alterations in lipid profiles
Nudotor et al. (2021) [15]	1,364 patients with T2DM	The study compared the efficacy of VSG and RYGB for long-term T2DM remission. The study used a retrospective cohort study design	T2DM remission, HbA1c less than 6.5%	Over a five-year follow-up period, it was discovered that RYGB was significantly more effective than VSG in achieving sustained remission of T2DM
Van Rijswijk et al. (2022) [16]	220 severely obese patients with T2DM	A randomized controlled clinical trial was undertaken to assess the comparative efficacy of two distinct bariatric surgical procedures: laparoscopic RYGB and laparoscopic OAGB, specifically concerning glycemic control	Glycemic control at 12 months follow-up, weight loss, surgical complications, psychologic status, and quality of life	Both RYGB and OAGB were effective in inducing rapid remission of T2DM in severely obese patients, as highlighted in the trial protocol
Du et al. (2018) [17]	58 class 1 obese patients with T2DM	Laparoscopic RYGB surgery, a minimally invasive weight loss and diabetes management treatment, was performed on all patients. The efficiency of laparoscopic RYGB in causing weight loss and T2DM remission was evaluated using outcome measures	Waist circumference, fasting plasma glucose levels, and outcomes of an OGTT at 2 hours	Over a 3-year follow-up period, laparoscopic RYGB was proven to be safe, efficacious, and practical in treating obese Chinese class I patients with T2DM
Zhang et al. (2020) [18]	35 participants who underwent RYGB and 70 who underwent SG	Participants who received RYGB were matched with up to two SG participants based on specified factors such as age, sex, body mass index, HbA1c level, medication use, duration of diabetes, and blood pressure. All surgical procedures, including SG and RYGB, were carried out laparoscopically	Rate of diabetes medication discontinuation, changes in serum cholesterol and low-density lipoprotein-c levels, and diabetes control within the 24-month follow-up period	Both RYGB and SG were great procedures for treating obesity in patients with type 2 diabetes; however, RYGB was linked to better outcomes than SG in terms of medication decrease related to metabolic disorders and dyslipidemia remission
Sahin et al. (2018) [19]	54 patients with T2DM	Participants who underwent the laparoscopic RYGB procedure had type 2 diabetes mellitus. The BMI of these individuals was then used to categorize them into groups: above or below 30 kg/m²	Changes in HbA1c levels, weight loss progression, and improvement in diabetes symptoms	The study questioned the suitability of cost-effectiveness analysis for non-obese overweight groups undergoing bariatric surgery, highlighting the need to prioritize patient outcomes and mortality reduction over economic considerations in such cases
van de pas et al. (2023) [20]	2,822 young adults and 24,497 adults who underwent RYGB or SG	A nationwide population-based cohort study utilizing data from the Dutch Audit Treatment of Obesity. Young adults (aged 18–25 years) and adults (aged 35–55 years) who underwent primary RYGB or SG were included	The primary outcome was the percentage total weight loss measured up to five years postoperatively. Secondary outcomes included the improvement of obesity-related comorbidities such as hypertension, dyslipidemia, and musculoskeletal pain	Bariatric surgery was found to be equally safe and effective in young adults as in adults, based on the study’s findings. Young adults showed superior weight loss outcomes compared to adults after undergoing RYGB or SG procedures
Guimaraes et al. (2021) [21]	601 patients who underwent RYGB	The study evaluated the long-term efficacy of RYGB in patients who underwent the procedure for obesity treatment	Percentage total weight loss, percentage excess weight loss, percentage excess BMI loss, rates of comorbidity remission, and complications associated with RYGB	RYGB is an effective long-term solution for weight loss and the improvement of obesity-related comorbidities, with a low incidence of serious complications
Sharples et al. (2019) [22]	52 patients with T2DM	The study reviewed the records of patients from a bariatric clinic who had a confirmed diagnosis of type 2 diabetes mellitus and underwent RYGB	Weight loss, HbA1c levels, medication use, and cost analysis	The study concluded that RYGB is effective in improving diabetic control, reducing medication use, facilitating significant weight loss, and providing substantial cost savings for obese patients with type 2 diabetes

Discussion

The studies reviewed offer a comprehensive evaluation of the effectiveness and safety of RYGB and SG in managing obesity and T2DM. van de Pas et al. (2023) conducted a large-scale study on young and older adults, demonstrating that bariatric surgery is equally safe and effective across age groups. Young adults achieved superior weight loss outcomes compared to adults, with significant improvements in obesity-related comorbidities such as hypertension, dyslipidemia, and musculoskeletal pain. This finding suggests that age should not be a limiting factor when considering candidates for bariatric surgery, as both young and older adults benefit substantially [20].

Comparative studies between RYGB and SG, such as those by Hage et al. (2024) and Zhang et al. (2020), highlight that RYGB generally offers higher efficacy for T2DM remission than SG. Hage et al. (2024) found that RYGB leads to better diabetes remission rates, reduced insulin use, and improved HbA1c levels compared to SG, regardless of patients’ baseline characteristics. Zhang et al. (2020) supported these findings, reporting that RYGB was more effective in reducing medication use and improving metabolic parameters such as serum cholesterol and low-density lipoprotein levels. These studies underscore the superiority of RYGB over SG in achieving more favorable metabolic outcomes in T2DM patients [11,18]. Several studies focused on the long-term efficacy of RYGB in managing T2DM and obesity. Girundi (2016) found that 87.6% of patients experienced T2DM remission 18 months post-RYGB, indicating significant long-term glycemic control. Similarly, Cheng et al. (2022) showed that RYGB was more effective than medical treatment alone in achieving and maintaining HbA1c levels below 6.5% over five years. Ji et al. (2020) and Du et al. (2018) extended these findings by demonstrating sustained weight loss, improved glycemic control, and positive changes in lipid profiles over multi-year follow-up periods, further establishing the long-term benefits of [12-14].

Giudicelli et al. (2023) and van Rijswijk et al. (2022) highlighted the importance of timely metabolic surgery for improving glycemic control and reducing cardiovascular risks in both young and older adults with T2DM. Giudicelli et al. (2023) emphasized that RYGB enhances metabolic health and diabetes outcomes, while van Rijswijk et al. (2022) compared RYGB and one-anastomosis gastric bypass, concluding that both procedures were effective in inducing rapid T2DM remission in severely obese patients. These studies illustrate the critical role of bariatric surgery in managing severe obesity and its associated comorbidities, regardless of the specific surgical procedure used [10,15].

Recommendations and future directions

To strengthen the evidence base, future research should prioritize randomized controlled trials (RCTs) that compare the effectiveness of RYGB and SG. RCTs can reduce selection bias and provide more robust conclusions about the comparative benefits and risks of these procedures. Developing and implementing standardized outcome measures across studies will facilitate easier comparisons and a clearer synthesis of results. Consistent reporting on key metrics such as percentage total weight loss, glycemic control, remission rates of T2DM, and improvement in obesity-related comorbidities is essential. This standardization will improve the overall quality of meta-analyses and systematic reviews. Future studies should extend the follow-up period beyond five years to assess the long-term sustainability of weight loss and metabolic improvements. This will provide a more comprehensive understanding of the durability of the benefits and the potential for late-onset complications. Research should include detailed analyses of postoperative nutritional monitoring and patient adherence to supplementation regimens. Investigating strategies to improve compliance with vitamin and mineral supplementation will help mitigate the risk of nutritional deficiencies.

Investigating the factors that predict success with different bariatric procedures can lead to more personalized treatment approaches. Identifying patient characteristics, such as genetic, metabolic, and behavioral factors, that influence outcomes can help tailor surgical options to individual needs. Personalized treatment plans can enhance the efficacy and safety of bariatric surgery for each patient.

## Conclusions

RYGB has been established as a practice in the treatment of T2DM and severe obesity. This systematic review underscores the various effects of RYGB, such as enhanced glycemic control, considerable and long-term weight loss, and reduced cardiovascular disease risks. The data in the present and previous work imply that RYGB is more efficient in diabetes remission and better influences metabolic profiles compared to conventional and intensive medical therapies; such advantageous impacts are applicable in young and elderly populations as well. However, the review also points toward the directions and the adverse effects of RYGB regarding metabolic and skeletal health. There are risks of nutritional deficiencies, increased fracture rates, and even diabetes relapse, which make patient selection, proper pre and postoperative investigation, and monitoring critical. Therefore, the lifelong necessity for the intake of supplements and the frequency of monitoring further complications relating to bone health are essential aspects to consider in patients who have undergone RYGB.

Further studies should be conducted on patient selection criteria to maximize the benefits of RYGB, post-surgery supportive care, and the genetic and molecular basis of the variability of RYGB outcomes. If all these challenges are managed, then the medical establishment will be able to optimize RYGB benefits concerning patients with T2DM and obesity. Therefore, RYGB is an effective treatment in the fight against T2DM and obesity, with the outcomes demonstrating improvements in metabolic and cardiovascular profiles. However, strategies that look at the advantages as well as drawbacks of various procedures are crucial for achieving the best outcomes for the patients.
